# 6K_2_-induced vesicles can move cell to cell during turnip mosaic virus infection

**DOI:** 10.3389/fmicb.2013.00351

**Published:** 2013-12-04

**Authors:** Romain Grangeon, Jun Jiang, Juan Wan, Maxime Agbeci, Huanquan Zheng, Jean-François Laliberté

**Affiliations:** ^1^Institut national de la recherche scientifique, INRS-Institut Armand-FrappierLaval, QC, Canada; ^2^Department of Biology, McGill UniversityMontréal, QC, Canada

**Keywords:** plant RNA virus, potyvirus, replication complex, membrane association, intercellular movement

## Abstract

To successfully infect plants, viruses replicate in an initially infected cell and then move to neighboring cells through plasmodesmata (PDs). However, the nature of the viral entity that crosses over the cell barrier into non-infected ones is not clear. The membrane-associated 6K_2_ protein of turnip mosaic virus (TuMV) induces the formation of vesicles involved in the replication and intracellular movement of viral RNA. This study shows that 6K_2_-induced vesicles trafficked toward the plasma membrane and were associated with plasmodesmata (PD). We demonstrated also that 6K_2_ moved cell-to-cell into adjoining cells when plants were infected with TuMV. 6K_2_ was then fused to photo-activable GFP (6K_2_:PAGFP) to visualize how 6K_2_ moved intercellularly during TuMV infection. After activation, 6K_2_:PAGFP-tagged vesicles moved to the cell periphery and across the cell wall into adjacent cells. These vesicles were shown to contain the viral RNA-dependent RNA polymerase and viral RNA. Symplasmic movement of TuMV may thus be achieved in the form of a membrane-associated viral RNA complex induced by 6K_2_.

## Introduction

Plant viruses encode movement proteins (MPs) that interact with plasmodesmata (PD) to achieve intercellular spread of virus infection (reviewed in Niehl and Heinlein, 2011). Several viral and host proteins that are involved in intra- and intercellular movement of plant viruses have been identified. These include MPs, host secretory pathway components and actomyosin motors (reviewed in Schoelz et al., 2011). However, the nature of the viral entity that crosses over the cell barrier of the infected cell into non-infected ones is not clear. For some icosahedral viruses, viral particles may transit through MP-induced tubules that go through PDs for their delivery into non-infected cells (van Lent et al., 1991; Pouwels et al., 2003; Amari et al., 2011; Chen et al., 2012). In the case of tobacco mosaic virus (TMV), a filamentous virus, it has been proposed that non-encapsidated infectious RNA molecules associated with MPs are being transported, possibly as an intact viral replication complex (VRC) (Kawakami et al., 2004). The exact composition of this RNA-protein complex has yet to be defined, in particular the contribution of other viral proteins and host components (e.g., proteins and lipids).

The genome of potyviruses is a single ~10 kb RNA molecule that codes for a polyprotein, which is processed into ten mature proteins. In addition to polyprotein-derived polypeptides, an ~7 kDa protein termed PIPO is produced in infected cells (Chung et al., 2008) and is also found as a trans-frame protein consisting of the amino-terminal half of P3 fused to PIPO (P3N-PIPO) (Vijayapalani et al., 2012). Potyviruses have no designated MP but many viral proteins have been reported to have MP-related functions. For instance, HCPro and the coat protein (CP) can increase the size exclusion limit (SEL) of PDs (Rojas et al., 1997). In addition, CP and the cylindrical inclusion (CI) protein are required for virus intercellular movement (Dolja et al., 1994, 1995; Carrington et al., 1998). These data showed that the core domain of CP provides a function essential during cell-to-cell movement and that the variable N- and C-terminal regions exposed on the virion surface are necessary for long distance transport. CI is further associated with PD, producing conical structures that extend through PD (Rodriguez-Cerezo et al., 1997; Roberts et al., 1998). The targeting of CI to PD is mediated by P3N-PIPO (Wei et al., 2010), which itself is targeted to the plasma membrane through an interaction with the host protein PCaP1 (Vijayapalani et al., 2012). The relationship of these viral proteins with viral RNA was, however, not investigated in the above studies.

One protein that may mediate the association of viral RNA with the above proteins is the 6K_2_ protein. TuMV infection leads to significant rearrangements of the early secretory pathway of the host cell. The infection is associated with the formation of at least two distinct types of sub-cellular compartments induced by the membrane-associated viral protein 6K_2_: a perinuclear globular structure and motile cortical vesicular structures (Grangeon et al., 2012). The perinuclear globular structure contains endoplasmic reticulum (ER), Golgi, COPII coatomers and chloroplasts. The motile vesicular structures are derived from the globular structure and move along transvacuolar and cortical ER. These vesicles contain viral RNA, replication viral proteins and host factors (Beauchemin and Laliberte, 2007; Beauchemin et al., 2007; Dufresne et al., 2008; Thivierge et al., 2008; Cotton et al., 2009; Huang et al., 2010). Latranculin B, which disrupts microfilaments, stops intracellular movement of 6K_2_ vesicles (Cotton et al., 2009), and TuMV intercellular movement (Agbeci et al., 2013). Thus, the 6K_2_ vesicles are involved in the movement of viral RNA.

Plant RNA viruses induce the remodeling of the secretory pathway (Schaad et al., 1997; Wei and Wang, 2008; Welsch et al., 2009; Cui et al., 2010; Bamunusinghe et al., 2011; Patarroyo et al., 2012; Linnik et al., 2013) or of organelles such as chloroplasts (Prod'homme et al., 2001, 2003; Jonczyk et al., 2007), mitochondria (Kopek et al., 2007; Hwang et al., 2008) and peroxisomes (McCartney et al., 2005) for viral replication. These “quasi-organelles” are often referred to as virus factories and harbor VRCs. Virus factories are, however, more than replication complexes associated with membranes. It has been suggested that replication and intercellular movement of some plant viruses are coordinated events that are mediated by membrane-associated motile vesicular structures (Tilsner and Oparka, 2012; Tilsner et al., 2012; Linnik et al., 2013). Recently, (Tilsner et al., 2013) showed the membrane-associated replication complex of potato virus X (PVX) are compartmentalized at PDs. In this study, we report that, during TuMV infection, 6K_2_ vesicles containing viral RNA and the viral RNA-dependent RNA polymerase (RdRp) were associated with PDs. Using photoactivable GFP fused to 6K_2_ (6K_2_:PAGFP), we observed activated 6K_2_:PAGFP vesicles crossing into adjacent cells by live cell imaging. These data suggest that the viral entity of TuMV that moves from cell-to-cell is associated with membranes that are recruited by 6K_2_.

## Materials and methods

### Molecular cloning and construction of fluorescent fusion proteins

The construction of pCambiaTuMV/6K_2_:GFP, pCambiaTuMV/6K_2_:mCherry, pCambia/6K_2_:PAGFP, CX:PAGFP, pGreen/RdRp:DsRed and pCambia/RdRp:GFP was described previously (Dufresne et al., 2008; Grangeon et al., 2012). The cloning of PDLP1:GFP and PDCB1:mCherry were described in (Amari et al., 2010, 2011). pCambia/6K_2_:mCherry was generated by a PCR amplification of 6K_2_ from pCambia/Tunos UK1 strain, using the following primer pairs (Forward: 5′-GCTCTAGAATGAACACCAGCGACATGA-3′; Reverse: 5′-CGGGATCCTTCATGGGTTACGGGTTCGGA-3′). The PCR product was digested with XbaI and BamHI and inserted into pCambia/mCherry (Beauchemin and Laliberte, 2007). The introduction of the 35S-GFP-HDEL gene cassette into pCambia/mCherry or pCambia/6K_2_:mCherry were done as follows. pBIN/20-ER-gk (Nelson et al., 2007) was digested with AseI and ligated with similarly digested pCambia/mCherry or pCambia/6K_2_:mCherry. Kanamycin-resistant *Escherichia coli* colonies were screened for either pCambia/mCherry/GFP-HDEL or pCambia/6K_2_:mCherry/GFP-HDEL. For pCambiaTuMV^VNN^, the Stratagene QuikChange II XL Multi Site-Directed Mutagenesis Kit and the primer pairs (Forward: 5′-CATCATCAGATTCTTCGTCAATGTAAATAATTTACTGCTAAGCGTACACCCA-3′; Reverse: 5′-TGGGTGTACGCTTAGCAGTAAATTATTTACATTGACGAAGAATCTGATGATG-3′) were used to create the mutation from p35Tunos (Cotton et al., 2009). The resulting mutant p35TuMV^VNN^ was digested with SmaI and ApaI, and then cloned into binary vector pCambia0390. The mutant was verified by sequencing.

### Protein expression in plants

All experiments were performed using *Nicotiana benthamiana*, an experimental TuMV host that supports the complete systemic infection cycle. The plants were grown in growth chambers under 16/8 h light/dark cycles, 24/20°C day/night temperatures. Transient expression studies were performed by agroinfiltration on 3-week-old *N. benthamiana* plants as described in (Grangeon et al., 2012). Plasmids were introduced by electroporation into *Agrobacterium tumefaciens* AGL1 strain and selected on LB ampicillin-kanamycin plates. Overnight cultures were diluted to an OD_600_ of 0.2 for pCambia/6K_2_:mCherry/GFP-HDEL, pCambia/mCherry/GFP-HDEL, pCambiaTuMV^VNN^, pCambia/6K_2_:PAGFP, pGreen/RdRp:DsRed, and pCambia/RdRp:GFP; 0.1 for CX:PAGFP, PDLP1:GFP, and PDCB1:mCherry for infiltration. For co-expression, 1:1 mixture of the two AGL1 bacteria containing the plasmids of interest was used for infiltration. Infiltrated plants were kept for 3–6 days post-agroinfiltration (dpa) in a growth chamber until observation. For FM4-64 staining, small pieces of *N. benthamiana* leaves were cut and dipped in 1 μg/μl of FM4-64 (Molecular Probes). Leaves were incubated at room temperature for 40–45 min and observed by confocal laser microscopy.

### Histological preparation and immunofluorescence labeling

One *N. benthamiana* leaf was agroinfiltrated with pCambiaTunos/6K_2_:GFP, and systemic infected leaves were collected at 6 day post-infiltration. Fixation, cell wall coloration with fluorescent brightener 28 (Calcofluor, Sigma-Aldrich), sucrose gradient and cryosectioning were processed as described (Knapp et al., 2012). Leaf cryosections were dried 2 h prior to the addition of PBS for 20 min. Cryosections were then incubated for 1 h in the blocking solution [phosphate-buffered saline (PBS), pH 7.4, containing 5% bovine serum albumin (BSA), 0.3% Triton X-100]. The cryosections were incubated for 1 h with mouse anti-dsRNA J2 antibody (1:500, English and Scientific Consulting Bt.) and washed three times with PBS for 10 min. The labeled cryosections were then incubated for 1 h with Alexa Fluor 568 goat anti-mouse IgG (1:500, Invitrogen), followed by washing four times with PBS for 10 min. SlowFade Gold (Molecular Probes) was mounted on the samples, and the coverslips were sealed with nail polish.

### Confocal microscopy

Agroinfiltrated leaf sections were mounted on a depression microscope slide, aligning the leaf tissue in the well. The cells were observed using a 10× objective, 40×, and/or 63× oil immersion objective on a LSM-780 confocal microscope (Zeiss). For LSM-780 microscope experiments, argon and HeNe lasers were used. Excitation/emission wavelengths were 488/495–540 nm for GFP, 561/600–630 nm for mCherry, 405/410–445 nm for Fluorescent Brightener 28, and 561 /570–640 nm for Alexa Fluor 568 goat anti-mouse IgG. Data from both green and red channels were collected at the same time. Photoactivation of GFP was done using ten to fifteen pulses of the 405-nm laser to activate PAGFP so that it produced very bright fluorescence emission that was detected by excitation at 488 nm using a 495- to 540-nm band pass filter. A 25-mW blue diode 405-nm laser was used at high output (50–100% transmission) to region in the cytoplasm using the photobleaching function of the Zeiss software in time-lapse mode. After acquisition, images were processed using ImageJ (1.46 k) and Carl Zeiss ZEN software. Area quantification of red fluorescence was performed using ImageJ. Statistical analysis was performed from a total of 15 leaf samples. Graphpad Prism Tukey's Multiple Comparison Test was used to assess whether the mean of two particular groups were different from each other. *P*-value summary (*P* < 0.05) shows statistically significant differences between different treatments.

## Results

### 6K_2_-induced vesicles reach the plasma membrane and plasmodesmata

**Figure 1:**
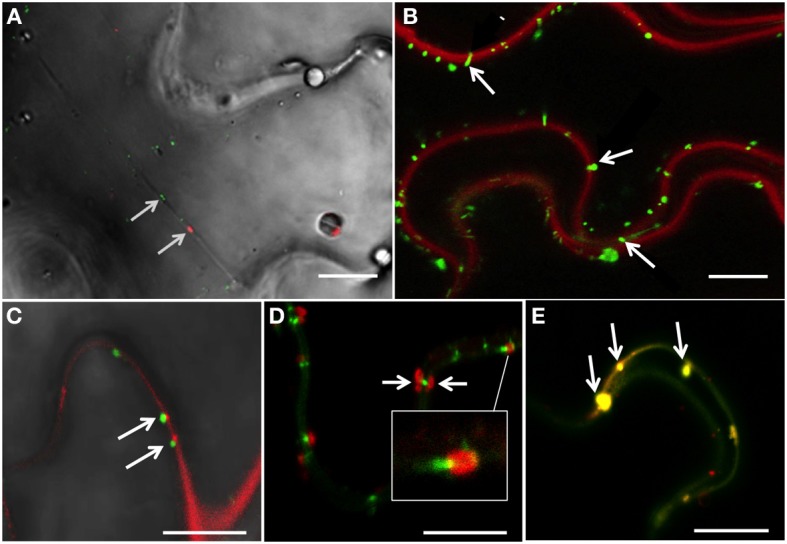
**6K_2_-induced vesicles reach the plasma membrane and plasmodesmata. (A)** Confocal image of a *N. benthamiana* cell infected with TuMV producing 6K_2_:mCherry and expressing PDLP1:GFP, with bright-field illumination. Arrows indicate the positioning of PDLP1:GFP and one 6K_2_ vesicle in a transvacuolar strand. **(B)** Confocal image of *N. benthamiana* cells infected with TuMV producing 6K_2_:GFP and stained with FM4-64 for plasma membrane labeling. Arrows indicate the presence of 6K_2_ vesicles anchored to the plasma membrane. **(C)** Confocal image of *N. benthamiana* cells infected with TuMV producing 6K_2_:GFP and expressing PDCB1:mCherry. Arrows indicate 6K_2_ vesicles located at PDCB1:mCherry-labeled PDs. **(D)** Confocal image of *N. benthamiana* cells infected with TuMV producing 6K_2_:mCherry expressing PDLP1:GFP. Arrows indicate 6K_2_ vesicles observed on either side of a PDLP1:GFP-labeled PD. White box is a close-up view of the structure indicated by white line. **(E)** Confocal image of *N. benthamiana* cells infected with TuMV producing 6K_2_:mCherry expressing PDLP1:GFP. Arrows indicate 6K_2_ vesicles located with PDLP1 within the intercellular wall space. **(A,B)** scale bar = 20μm. **(C–E)** scale bar = 10μm.

The protein 6K_2_ of TuMV is an integral membrane protein (Beauchemin et al., 2007) involved in endomembrane rearrangements for the generation of viral compartments important for replication and intracellular movement (Cotton et al., 2009; Grangeon et al., 2012). TuMV-induced 6K_2_-tagged vesicles move along actin microfilaments (Cotton et al., 2009) but the final destination of this intracellular movement was not determined. When *N. benthamiana* plants were infected with TuMV that produced 6K_2_:mCherry-tagged vesicles (Agbeci et al., 2013) and co-expressed with the Plasmodesmata Located Protein 1 (PDLP1) fused to GFP (Thomas et al., 2008), both PDLP1:GFP and 6K_2_:mCherry-tagged vesicles were found to traffic in transvacuolar cytoplasmic strands, which were visualized by bright-field illumination (Figure 1A and Supplemental Movie S1). Transvacuolar cytoplasmic strands connect the nuclear region with the cell periphery (Reisen et al., 2005) suggesting that 6K_2_ vesicles have the potential to reach the plasma membrane.

We stained *N. benthamiana* leaves infected with TuMV producing 6K_2_:GFP-tagged vesicles with the red FM4-64 dye used to label the plasma membrane (Bolte et al., 2004) to examine if any of 6K_2_-tagged vesicles were associated with this membrane. Numerous 6K_2_:GFP-tagged vesicles were observed at the plasma membrane (Figure 1B, arrows). To determine if 6K_2_-tagged vesicles were located at PDs, plants were infected with TuMV producing 6K_2_:GFP vesicles and co-expressed with the Plasmodesmata Callose Binding Protein 1 (PDCB1) fused to mCherry (Amari et al., 2011). 6K_2_:GFP tagged vesicles were found adjacent to PDs labeled with PDCB1:mCherry (Figure 1C, arrows). TuMV-induced 6K_2_:mCherry-tagged vesicles were also found in association with PDLP1:GFP (Figure 1D, white box) and in several instances 6K_2_ vesicles were present at the opposite ends of PDLP1-labeled PD (Figure 1D, arrows). 6K_2_-tagged vesicles were also found to localize with PDLP1 within the intercellular wall space (Figure 1E, arrows). These data suggest that 6K_2_ vesicles move intracellularly toward the plasma membrane and can be associated with PDs.

**Figure 2:**
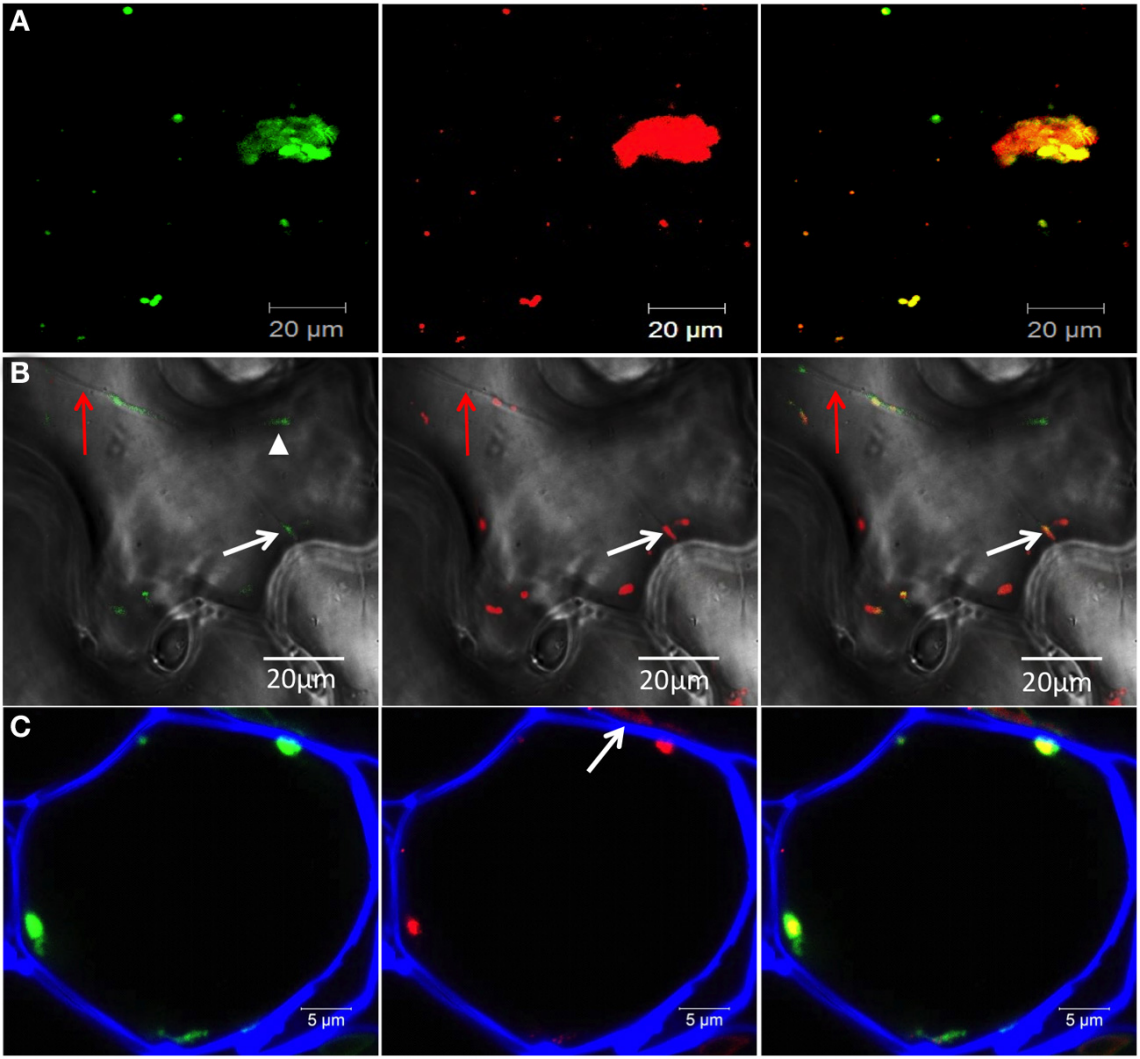
**6K_2_-induced vesicles contain RdRp and viral RNA. (A,B)** Confocal images of leaf epidermal cells of *N. benthamiana* showing RdRp:GFP (left panel) in TuMV-infected cells producing 6K_2_:mCherry-tagged vesicles (middle panel) and merged data (right panel). Images shown in **(A)** are three-dimensional projections of >30 1-μ m-thick slices that overlap by 0.5 μm. Images shown in **(B)** are single optical images with bright-field illumination. Red arrow indicates a transvacuolar strand, white arrow shows a 6K_2_-induced vesicle containing RdRp close to the plasma membrane and arrow head shows RdRp:GFP not associated with 6K_2_:mCherry **(C)** Cryo-histological section of a parenchymal cell from a leaf systemically infected with TuMV producing 6K_2_:GFP vesicles (left panel). Middle panel shows immunostaining with a mouse anti-dsRNA monoclonal antibody and an Alexa Fluor 568 conjugated goat anti-mouse IgG (in red). Right panel is a merger of left and middle panels. Cell wall is shown in blue. **(A,B)** scale bar = 20μm. (C) scale bar = 5 μm.

We investigated if motile 6K_2_ vesicles, especially those in the vicinity of the plasma membrane, contained the TuMV RNA-dependent RNA polymerase (RdRp) important for replication. *N. benthamiana* plants were infected with TuMV producing 6K_2_:mCherry-tagged vesicles and co-expressed with the viral RdRp fused to GFP (RdRp:GFP). We found that of the 26 motile 6K_2_:mCherry vesicles that were counted in Figure 2A, 22 were associated with RdRp:GFP fluorescence, suggesting the presence of RdRp in these structures. As expected, co-localization of red and green fluorescence was found in the large perinuclear structure. We also looked at 6K_2_ vesicles near the cell border (shown by bright-field illumination) and found them to be associated with RdRp (Figure 2B, white arrow). A population of RdRp is also present in a soluble form in infected cells (Cotton et al., 2009) and may explain why the RdRp:GFP structure denoted by a arrow head in Figure 2B does not localize with membrane-bound 6K_2_:mCherry. We also determined if 6K_2_ vesicles contained viral RNA. Cryo-histological sections of mock and TuMV/6K_2_:GFP systemically infected leaves were stained with a monoclonal antibody that recognizes double-stranded (ds) RNA and then observed by confocal microscopy. Figure 2C (left panel) shows the distribution of 6K_2_ vesicles near the cell wall in a parenchymal cell. The cell wall was stained with Fluorescent Brightener 28 and shown in blue. We found that there was some non-specific labeling, often in irregular shape, with the anti-dsRNA monoclonal antibody in mock- (not shown) and TuMV-infected cells (arrow, Figure 2C). Nonetheless, all 6K_2_:GFP vesicles with regular sphere shape were positive for dsRNA. Several other histological sections were examined and 6K_2_ vesicles were always positive for dsRNA. Furthermore, the intensity of the red signal was proportional to the intensity of the green signal, indicating the specificity of the labeling with the monoclonal antibody of the 6K_2_ vesicles. These data indicate that 6K_2_ vesicles in proximity of the plasma membrane and at PDs are potentially active for replication.

### 6K_2_ moves intercellularly only when cells are infected with TuMV

**Figure 3:**
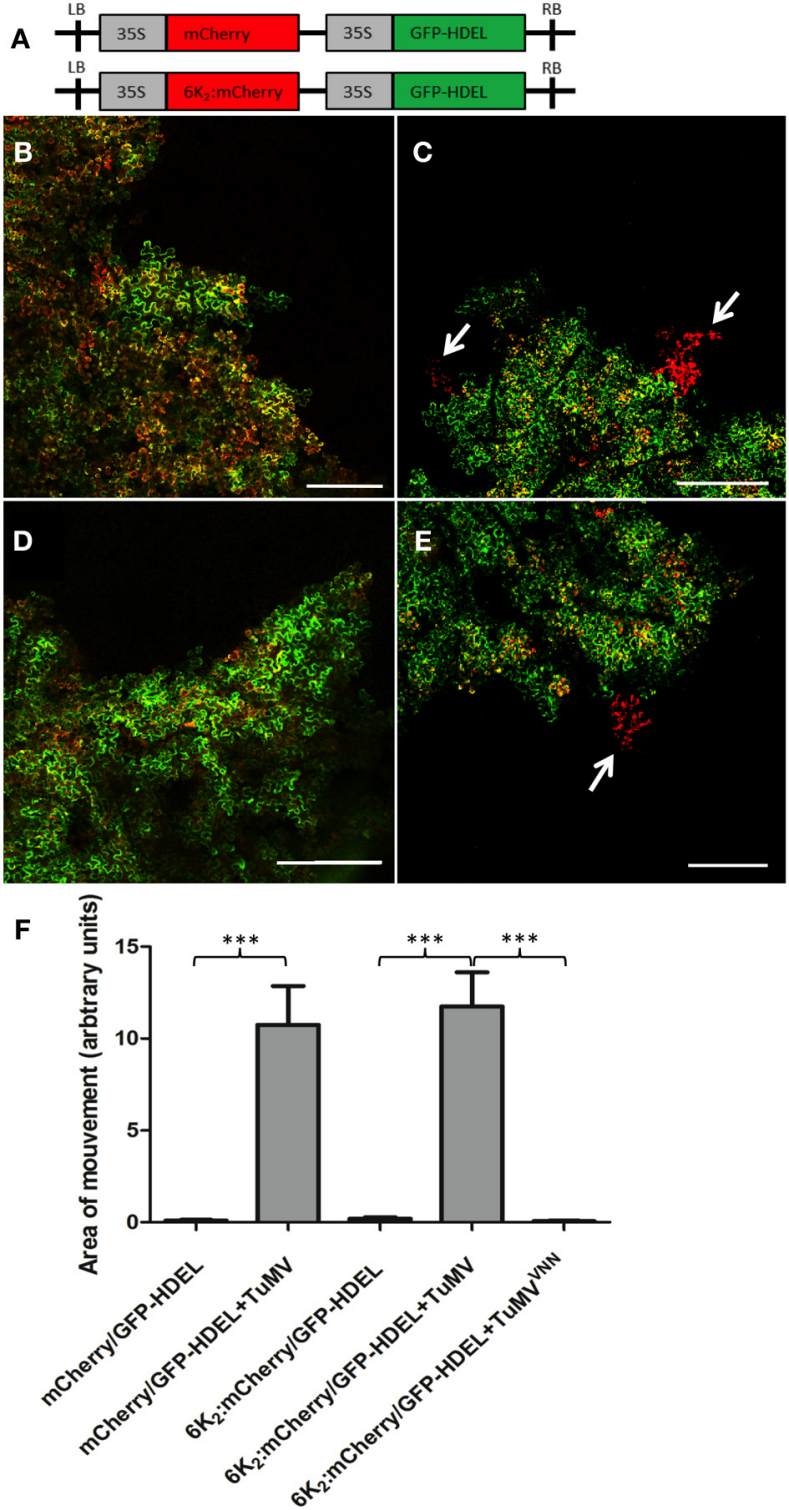
**6K_2_ moves intercellularly only when cells are infected with TuMV. (A)** Schematic representation of the pCambia/mCherry/GFP-HDEL and pCambia/6K_2_:mCherry/GFP-HDEL. **(B,C)** Confocal images of cells expressing pCambia/mCherry/GFP-HDEL in mock- **(B)** or TuMV-infected **(C)** *N. benthamiana* plants. **(D,E)** Confocal images of cells expressing pCambia/6K_2_:mCherry/GFP-HDEL in mock- **(D)** or TuMV-infected **(E)** *N. benthamiana* plants. Arrows indicate areas of mCherry or 6K2:mCherry protein movement. **(F)** Area of movement has been quantified for 15 *N. benthamiana* epidermal samples expressing the indicated proteins. Brackets and asterisks indicate statistical differences as follows: ^***^P-value < 0.001 (extremely significant). Scale bars = 500 μm.

To determine if TuMV 6K_2_ can move cell-to-cell, we introduced a gene cassette encoding GFP-HDEL under the control of the cauliflower mosaic virus (CaMV) 35S promoter next to either a mCherry- or a 6K_2_:mcherry-encoding cassette within the left and right borders of the T-DNA in the binary vector pCambia (pCambia/mCherry/GFP-HDEL or pCambia/6K_2_:mCherry/GFP-HDEL) (Figure 3A). We showed previously that GFP-HDEL does not move between cells during TuMV infection (Agbeci et al., 2013). Because both gene cassettes are delivered into the same cells, agroinfiltrated cells are characterized by concomitant green and red fluorescence and any intercellular movement of the mCherry fusion would be characterized by the presence of red-only fluorescence. At 6-day post-agroinfiltration with pCambia/mCherry/GFP-HDEL or pCambia/6K_2_:mCherry/GFP-HDEL, all cells emitted both green and red fluorescence when observed under the confocal microscope at low magnification (Figures 3B,D). No red-only fluorescence foci were observed, indicating that either mCherry or 6K_2_:mCherry did not move out by diffusion from agroinfiltrated cells into adjacent cells. When pCambia/mCherry/GFP-HDEL or pCambia/6K_2_:mCherry/GFP-HDEL were expressed with a non-fluorescent TuMV infectious clone, in addition to cells emitting both red and green fluorescence, cells emitting only red fluorescence were observed (Figures 3C,E). Quantitative analysis of intercellular movement was performed by measuring surface area of red fluorescence-only foci of 15 leaf samples and was shown to be statistically significant (Figure 3F). No intercellular movement of 6K_2_:mCherry was observed when the dual gene cassette was expressed along with a non-replicative TuMV cDNA clone in which the GDD motif of the RdRp was replaced by a VNN motif (Figure 3F). It was previously demonstrated that MPs move into adjacent cells during a virus infection (Fujiwara et al., 1993). The above experiment showed that the 6K_2_ membrane protein, in the presence of TuMV infection, behaved similarly.

### 6K_2_-tagged vesicles move intercellularly

**Figure 4:**
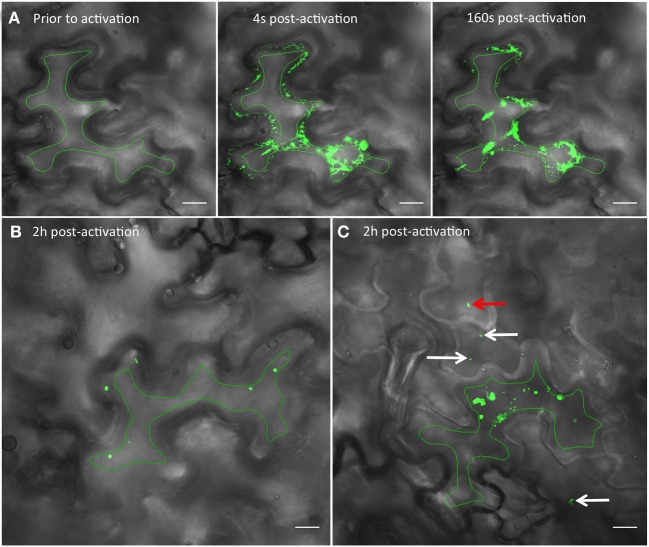
**Photoactivation of PAGFP shows intercellular movement of 6K_2_ vesicles. (A)** Time series images showing a *N. benthamiana* cell expressing the ER protein calnexin fused to PAGFP (CX:PAGFP) prior to activation (left panel), 4 (middle panel), or 160 sec (right panel) after activation using a 405 nm diode. **(B)** Non-infected cell expressing 6K_2_:PAGFP 2h after photoactivation. **(C)** Cell infected with TuMV producing 6K_2_:mCherry-tagged vesicles and expressing 6K_2_:PAGFP 2h after photoactivation. The red channel is not represented. White arrows in **(C)** point to vesicles that have moved through one cell layer. Red arrow in **(C)** points to a vesicle that has moved through two cell layers. Green line in **(A–C)** indicates activation area. Scale bar = 20μm.

To determine if TuMV 6K_2_-tagged vesicles can move into neighboring cells, we fused 6K_2_ with photoactivable GFP (PAGFP), which is widely used in fluorescent pulse labeling to track the cellular distribution of proteins or organelles from a specific position within a cell (Runions et al., 2006). First, to ascertain that PAGFP was activated only within a single cell, the ER protein calnexin fused to PAGFP (CX:PAGFP) (Runions et al., 2006) was expressed by agroinfiltration in *N. benthamiana* cells. A single epidermal cell expressing CX:PAGFP was activated and the activated CX:PAGFP distribution was followed by time-lapse microscopy (Figure 4A). No green fluorescence was observed prior to activation (Figure 4A, left panel) but the signal in the ER was clearly noticeable right after activation, with no signal observed in the adjoining cells (Figure 4A, middle panel). ER labeling was still strong 160 sec after activation and no fluorescence was monitored in the adjoining cells (Figure 4A, right panel). We repeated this experiment ten times without observing any green fluorescence in the adjoining cells, even after a 5 min observation period. This indicated that PAGFP activation occurred only in the targeted cell and not in the neighboring cells.

**Figure 5:**
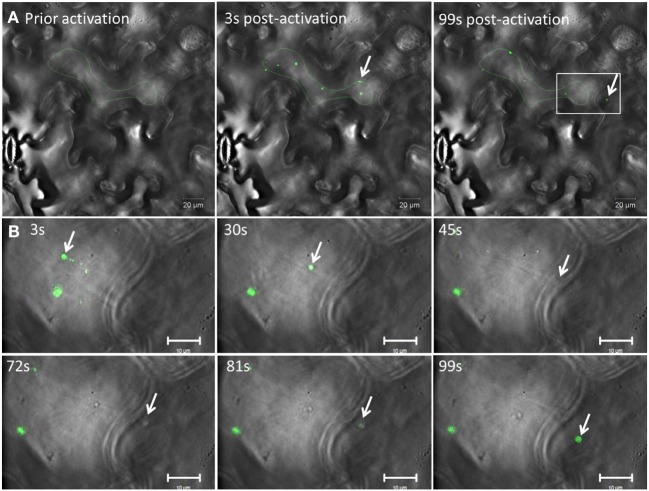
**TuMV-induced 6K_2_-tagged vesicle moves intercellularly. (A)** Time series images of a photoactivated cell infected with TuMV producing 6K_2_:mCherry-tagged vesicles and expressing 6K_2_:PAGFP. The red channel is not represented. Left panel shows a cell prior to photoactivation, middle panel shows a cell 3 sec after activation using a 405 nm diode. Right panel shows a cell 99 sec after activation. Green line indicates activation area. **(B)** Close-up view of a square depicted in the right panel in **(A)** from 3 to 99 sec after activation. The panels show the position of an activated 6K_2_:PAGFP vesicle (arrow) at the indicated time. In the 45 s panel, vesicle is not seen in green fluorescence because of the loss of the focal plane, but was faintly noticeable in bright field (see Supplemental movie S2). **(A)** scale bar = 20μm, **(B)** scale bar = 10μm.

We then used 6K_2_:PAGFP to monitor 6K_2_ vesicle intercellular movement in mock- or TuMV-infected cells. We first activated 6K_2_:PAGFP in 30 different cells in mock-infected *N. benthamiana* leaves and we monitored for the presence of 6K_2_:PAGFP-activated vesicles in neighboring cells 2 h after activation (Figure 4B). In all cases and in accordance to the data presented above, no vesicle was observed in the adjoining cells. We then activated 6K_2_:PAGFP in 39 different TuMV-infected cells producing 6K_2_:mCherry-tagged vesicles and we found that 62% of the activated cells (24/39 cells) had vesicles that crossed over into adjoining cells. Figure 4C shows a representative activated cell where 3 vesicles (white arrows) were observed in 2 neighboring cells and also one vesicle that probably traveled through 2 cell layers (red arrow) (the red channel is not represented for better visualization of the green signal). We also followed intercellular movement of 6K_2_ vesicles in TuMV-infected cells producing 6K_2_:mCherry-tagged vesicles and expressing 6K_2_:PAGFP in real time. Figure 5A shows an activated green fluorescing motile 6K_2_ vesicle (designated by the arrow) that moved rapidly into the adjacent cell (the red channel is no represented). Figure 5B and Supplemental Movie S2 detail how this vesicle crossed into the adjacent cell. This vesicle trafficked in a transvacuolar strand toward the cell periphery, which then moved out of the fluorescent focal plane for a few seconds through the cell wall to reappear back in the focal plane in the adjacent cell. Movement of the vesicle can partially be followed looking at the bright field. This vesicle moved for a distance of at least 70 μm after activation, with an average velocity of 0.3 μm/sec. These experiments provide direct evidence that 6K_2_ vesicles can move cell to cell, and that this process required viral infection.

**Figure 6:**
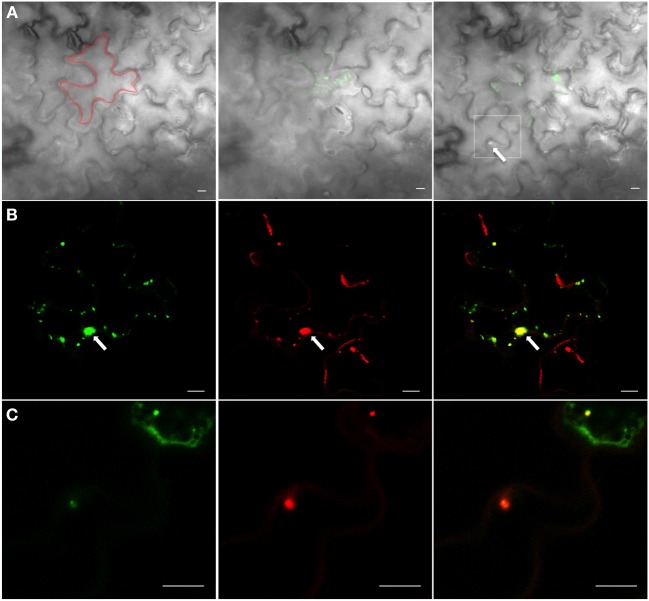
**6K_2_-induced vesicles that have moved intercellularly contain RdRp. (A)** Cells infected with TuMV expressing RdRp:DsRed and 6K_2_:PAGFP before activation (left panel), at activation (middle panel), and 2 h after activation (right panel). Only the green signal is shown. The bright field indicates cell borders; Red line indicates activation area; white square denotes the presence of an activated 6K2:PAGFP vesicle in adjoining cell **(B)** Activated 6K_2_:PAGFP in a cell from the middle panel of **(A)** (left panel), along with signal from RdRp:DsRed (middle panel). Right panel is the merger of the left and middle images. **(C)** An activated 6K_2_:PAGFP vesicle that has moved into neighboring cell shown in white square in right panel of **(A)** (left panel), along with signal from RdRp:DsRed (middle panel). Right panel is the merger of the left and middle data. Scale bar = 10μm.

We then determined whether activated 6K_2_:PAGFP vesicles that moved into adjoining cells were empty structures or still contained replication components, such as the RdRp. 6K_2_:PAGFP was co-expressed with RdRp:DsRed in TuMV-infected cells. 6K_2_:PAGFP was then activated in a TuMV-infected cells (area of activation is highlighted by a red line, Figure 6A). Presence of a red-labeled perinuclear structure (arrow, Figure 6B) was the marker used to ascertain that the cell was infected with TuMV. At activation, most of the activated 6K_2_:PAGFP vesicles were positive for RdRp:DsRed (Figure 6B). Two hours later, one activated 6K_2_:GFP was seen in a neighboring cell (Figure 6A, right panel, arrow in the white square). This vesicle was positive for RdRp-DsRed (Figure 6C). This experiment was repeated 15 times, and similar results were observed. This experiment suggests that the 6K_2_ vesicles that move intercellularly are likely to be replication competent.

## Discussion

There are increasing data indicating that plant VRCs move at least as far as the cell wall via membrane-bound vesicles. VRCs recruitment by membrane-associated MPs and the presence of this complex at the cell periphery has been reported for TMV, PVX and red clover necrotic mosaic virus (Kawakami et al., 2004; Kaido et al., 2011; Tilsner et al., 2012). Recently, replication and trafficking of PVX have been shown to be coupled at the entrances of PDs (Tilsner et al., 2013). Hence, MPs may capture VRCs at PD, suggesting a link between viral RNA replication and movement (Tilsner and Oparka, 2012). This vesicle transport is reminiscent of the pattern of viral egress reported for animal viruses (reviewed in Den Boon and Ahlquist, 2010).

This study takes this area of investigation further and points to a route for plant membrane-bound VRCs that go beyond the cell wall. TuMV infection is associated with the remodeling of the endomembrane system and formation of large perinuclear structures that leads to the release of motile viral vesicles (Grangeon et al., 2012). These vesicles are considered to be the site where virus replication takes place (Schaad et al., 1997; Cotton et al., 2009; Grangeon et al., 2012). We show in this study that TuMV-induced 6K_2_-tagged vesicles could carry this viral RNA-protein cargo to PDs. First, 6K_2_ vesicles moved in transvacuolar cytoplasmic strands [Figure 1A, Supplemental Movie S1 and (Grangeon et al., 2012)] to reach the plasma membrane (Figure 1B). The Triple Gene Block proteins 2 and 3 of potato mop-top virus as well as the *beet yellows virus* Hsp70 homolog have also been shown to move in these strands to reach the cell periphery and PDs (Cowan et al., 2002; Prokhnevsky et al., 2005). Importantly, 6K_2_ vesicles were found to be associated with PDs (Figures 1C–E). The targeting of CI to PD was shown to be mediated by P3N-PIPO (Wei et al., 2010), which itself is targeted to the plasma membrane through an interaction with the host protein PCaP1 (Vijayapalani et al., 2012). The CI protein has been found to be associated with 6K_2_ vesicles (Cotton et al., 2009), which may be the factor that contributes to PD localization of 6K_2_ vesicles.

Furthermore, 6K_2_ was shown to be able to move intercellularly during TuMV infection (Figure 3). The SEL of PD is normally too small to allow the passive transit of a virus complex (Zambryski, 1995). MPs are known to increase the SEL of PD, allowing the cell-to-cell spread of much larger molecular complexes (Wolf et al., 1989; Deom et al., 1990; Poirson et al., 1993). For potyviruses, it was shown that HC-Pro and CP increased PD SEL (Rojas et al., 1997). Consequently, the SEL increase during TuMV infection allowed cell-to-cell movement not only of soluble individual proteins but also of 6K_2_, which moved intercellularly as vesicles, as shown by live cell imaging (Figures 4, 5, and Supplemental Movie S2).

The vesicles that move intercellularly are apparently not empty structures. With very few exceptions, 6K_2_ vesicles were shown to be positive for the presence of RdRp and viral RNA (Figure 2), and vesicles that have been shown to move into neighboring cells also contained RdRP (Figure 6). The presence of theses viral components in 6K_2_ vesicles is also supported by previous studies, which showed that several viral proteins and host proteins, in addition to the viral RNA, have been found within 6K_2_-induced structures (Beauchemin and Laliberte, 2007; Beauchemin et al., 2007; Dufresne et al., 2008; Cotton et al., 2009; Cui et al., 2010). This suggests that the viral entity of TuMV that transits into non-infected cells is a highly intricate RNA-protein complex bounded by a lipid membrane. The exact composition of this RNA-protein complex needs, however, to be determined. The histological data showed that 6K_2_ vesicles contained dsRNA, but demonstrating that viral RNA is associated with a 6K_2_ vesicle that has moved into a neighboring cell is technically challenging. Additionally, the 6K_2_ vesicles might have acquired the RdRp in the target cell because RdRp:dsRed is expressed in trans and in the entire zone from agrobacteria. In a previous study, we showed by immunocytochemistry that viral RNA punctate structures are associated by RdRp in infected protoplasts (Cotton et al., 2009). At the moment, only 6K_2_ has been shown to be associated with the viral RNA, but we cannot exclude that the viral RNA moves cell-to-cell complexed with yet-to-be defined factors in a manner that do not involve 6K_2_. Another element will be to investigate the relationship of CP, CI and P3N-PIPO with 6K_2_ to see how these proteins work in concert for moving 6K_2_ vesicles into neighboring cells.

Kawakami et al. (2004) proposed similar transport mode for TMV, which moved intercellularly as intact VRCs associated with MP membrane bodies. Additionally, the presence of PVX RNA associated with the membrane-associated TGB1 protein within PDs was shown (Tilsner et al., 2013). These investigations support the idea that for certain of plant viruses, the viral RNA can move cell-to-cell in a membrane associated structure.

## Author contributions

Romain Grangeon, Jun Jiang and Juan Wan designed and performed the experiments, analyzed the data and wrote the manuscript. Maxime Agbeci provided reagents. Huanquan Zheng and Jean-François Laliberté conceived the experiments and wrote the manuscript.

### Conflict of interest statement

The authors declare that the research was conducted in the absence of any commercial or financial relationships that could be construed as a potential conflict of interest.

## Supplementary material

Supplemental movie S1 **Times lapse experiment of *N. benthamiana* cell infected with TuMV producing 6K_2_:mCherry-tagged vesicles and expressing PDLP1:GFP, with bright-field illumination.** PDLP1:GFP and one 6K_2_ vesicle can be seen trafficking on a transvacuolar strand. Images were acquired every 4 sec using the 40X objective of a LSM 780 confocal microscope.

Supplemental movie S2 **Time-lapse images of a photoactivated cell infected with TuMV producing 6K_2_:mCherry-tagged vesicles and expressing 6K_2_:PAGFP.** Images were acquired every 3 sec using the 40X objective of a LSM 780 confocal microscope.
